# Spawning Sites of the Japanese Eel in Relation to Oceanographic Structure and the West Mariana Ridge

**DOI:** 10.1371/journal.pone.0088759

**Published:** 2014-02-13

**Authors:** Jun Aoyama, Shun Watanabe, Michael J. Miller, Noritaka Mochioka, Tsuguo Otake, Tatsuki Yoshinaga, Katsumi Tsukamoto

**Affiliations:** 1 Atmosphere and Ocean Research Institute, The University of Tokyo, Chiba, Japan; 2 College of Bioresource Sciences, Nihon University, Kanagawa, Japan; 3 Faculty of Agriculture, Kyushu University, Fukuoka, Japan; 4 School of Marine Biosciences, Kitasato University, Kanagawa, Japan; Institut Maurice-Lamontagne, Canada

## Abstract

The Japanese eel, *Anguilla japonica*, spawns within the North Equatorial Current that bifurcates into both northward and southward flows in its westward region, so its spawning location and larval transport dynamics seem important for understanding fluctuations in its recruitment to East Asia. Intensive research efforts determined that Japanese eels spawn along the western side of the West Mariana Ridge during new moon periods, where all oceanic life history stages have been collected, including eggs and spawning adults. However, how the eels decide where to form spawning aggregations is unknown because spawning appears to have occurred at various latitudes. A salinity front formed from tropical rainfall was hypothesized to determine the latitude of its spawning locations, but an exact spawning site was only found once by collecting eggs in May 2009. This study reports on the collections of Japanese eel eggs and preleptocephali during three new moon periods in June 2011 and May and June 2012 at locations indicating that the distribution of lower salinity surface water or salinity fronts influence the latitude of spawning sites along the ridge. A distinct salinity front may concentrate spawning south of the front on the western side of the seamount ridge. It was also suggested that eels may spawn at various latitudes within low-salinity water when the salinity fronts appeared unclear. Eel eggs were distributed within the 150–180 m layer near the top of the thermocline, indicating shallow spawning depths. Using these landmarks for latitude (salinity front), longitude (seamount ridge), and depth (top of the thermocline) to guide the formation of spawning aggregations could facilitate finding mates and help synchronize their spawning.

## Introduction

Freshwater eels of the genus *Anguilla* are catadromous fishes that spawn over deep water at tropical latitudes and use the ocean for their larval development before entering estuarine and freshwater growth habitats [1], [2]. All three northern temperate species of anguillid eels consist of single panmictic populations [3]–[7], with all of their reproductively maturing individuals migrating long distances offshore to spawn in a single spawning area for each species [2], [8].

**Figure 1:**
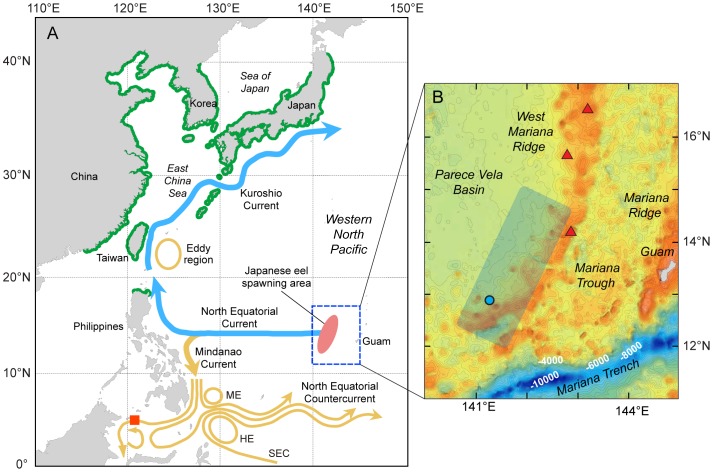
The Japanese eel, *Anguilla japonica*, spawning area in the western North Pacific region. Japanese eel leptocephali are transported from their spawning area (light-red oval) by the North Equatorial Current (NEC) into the Kuroshio Current (blue lines), which transports them towards waters near their recruitment areas where they metamorphose into glass eels (A). Recruitment areas of *A. japonica* are shown with green lines on coastlines (inland habitats not shown), and currents or eddies shown in light-brown are inappropriate or disadvantageous pathways for leptocephali. The location where a 42.8 mm *A. japonica* leptocephalus was collected in the Celebes Sea outside of its normal recruitment region is shown with a red square. The blue rectangle shows the areas of the other maps (Fig. 2,4), and the South Equatorial Current (SEC), Halmahera Eddy (HE), Mindanao Eddy (ME) and other currents are also shown. The left panel (B) shows the bathymetric structure of the West Mariana Ridge and that preleptocephali were only collected on the western side of the southern region of the ridge in previous surveys (within the blue shaded area) from 2005–2009 and eggs were only collected in one area in 2009 (blue circle) [19].

Anguillid eel populations including those of the Japanese eel, *Anguilla japonica*, have declined worldwide in recent decades [9], [10], but the exact causes of the declines are difficult to determine partly because their reproductive ecology is hidden by the vast open ocean. The spawning areas of the Atlantic eels, the European eel, *Anguilla anguilla*, and the American eel, *Anguilla rostrata*, were discovered early in the last century [11] and were later found to be associated with temperature fronts in the Sargasso Sea based on the distribution of small larvae [12], [13], with spawning occurring across a wide latitudinal area [14], [15]. The spawning area of the Japanese eel in the western North Pacific (Fig. 1) was discovered in 1991 [16] and has been intensively studied in the last few decades [17]–[19]. The surveys that succeeded to collect newly hatched larvae [19]–[21], eggs and spawning-condition adults [19], [21], [22] indicated that spawning area of the Japanese eel is located latitudinally from about 12–15°N [17]–[19] within the continuous westward flow of the North Equatorial Current (NEC) that is present from about 8–17°N [23], [24], and longitudinally along the western side of the West Mariana Ridge, which is the southwestern extension of the Izu-Bonin-Mariana Arc system (see Gardner [25]). Further, otolith analyses for the larvae collected during the surveys showed that spawning of Japanese eels occur during new moon periods [17], [26].

Various types of research have been conducted recently to understand the oceanic life histories of anguillid eels. The physiological ecology of migration [27], [28], biology of maturation and spawning behavior [29]–[31], or geomagnetic sense [32] has been studied in the laboratory. Pop-up satellite transmitting tags have been used to learn about the migratory behavior of both Northern [33], [34] and Southern [35]–[37] Hemisphere anguillids. These studies suggest that anguillid silver eels have incredible long-term swimming abilities that might be guided in part by a geomagnetic sense while they migrate through the ocean using distinct diel vertical migration behaviors. Exactly how they find their spawning areas and decide where to form spawning aggregations has remained a mystery however [31] and spawning eels have not yet been observed directly [38].

A shallow salinity front that forms within the NEC where Japanese eels spawn [14], [34] (Fig. 1) has been hypothesized to affect the latitude of spawning [39], [40]. Indeed, collections of small leptocephali [39] or eggs [19] were made near the southern edge of the spawning area when the salinity front was located far to the south in two different years. Larger leptocephali were also found south of the salinity front further to the west during their larval transport [16]. However, in some years there are no distinct salinity fronts in the spawning area [17], [41], so what determines spawning locations at those times has remained unclear.

Understanding how Japanese eels decide their latitude of spawning is of special importance because the NEC bifurcates into both northward and southward flows (Fig.1). The latitude of bifurcation of the two current flows can change in different months or years [42]–[44], which may strongly affect how many Japanese eel larvae get entrained into the southward flowing Mindanao Current and transported away from their recruitment areas [43], [45]. Therefore, at what latitude the eels decide to spawn could have a significant affect on the recruitment success of their larvae [40], [43], [45].

This study analyzes the results of three sampling surveys for eggs and pre-feeding stage preleptocephali in 2011 and 2012 that were conducted to learn more about what factors may determine where Japanese eels form spawning aggregations. The three sampling surveys were designed to determine the distribution of eggs and preleptocephali in the NEC along the ridge during new moon periods to evaluate where spawning may have occurred and where it did not occur by the same set of protocols. Because the temperature structure of the warm surface layer of the NEC does not include any distinct gradients or fronts at the latitudes where the Japanese eel spawns [23], [41], [46], the salinity structure was evaluated in relation to where eggs and larvae were collected. This information along with the findings of previous studies is used to propose a hypothesis for where this species will spawn along the seamount ridge.

## Materials and Methods

### Survey Strategy of Cruises

The three cruises of this study were conducted during 24 June–10 July in 2011 (KH-11-6), and 13 May–1 June (leg 1 of KH-12-2) and 6 June–28 June 2012 (leg 2 of KH-12-2, Table 1). Oceanographic observations were made at the beginning of each cruise to particularly know the location of the salinity front as it crossed the seamount chain before deciding where to sample for eggs just before new moon. Based on the salinity structure, a region along the ridge was chosen for sampling for eggs just before new moon, and an arrangement of stations was set along the west side of the ridge. These stations were sampled until eggs were collected, and then a new grid of stations was arranged around the location of the first egg collection, which was intensively sampled until the eggs would likely be hatching into preleptocephali. Then transect surveys were conducted to find newly hatched preleptocephali at different latitudes along the ridge to estimate where else spawning may have occurred during each new moon period. These transects were designed to detect spawning near the ridge based on the presence of preleptocephali, but were not extensive enough to exclude spawning in other areas with certainty if preleptocephali were not collected if there were longitudinal variations in where spawning may have occurred in relation to the ridge.

**Table 1:** Overview of the number of stations sampled during the different parts of each of the three surveys in relation to the timing of new moon and the number of eggs or preleptocephali that were collected in 2011 (KH-11-6, new moon 1 July) and 2012 (KH-12-2: leg 1, new moon 20 May 2; leg 2, new moon 19 June).

Cruise	Pre-survey		Egg grid survey			Preleptocephalus survey		
	Period	No. of stations	Period	No. of stations (tows)	No of eggs collected	Period	No. of stations	No. of prelepto. collected
KH-11-6	28 June	3	29–30 June	9 (17)	147	1–6 July	46	83
KH-12-2 leg 1	18 May	8	18–20 May	14 (15)	131*	20–25 May	25	153
KH-12-2 leg 2	14–15 June	13	16–19 June	29 (45)	284	19–23 June	37	47

*4 eggs were collected on 21 May (one day after New moon) after the egg grid survey.

### Locating the Salinity Front

**Figure 2:**
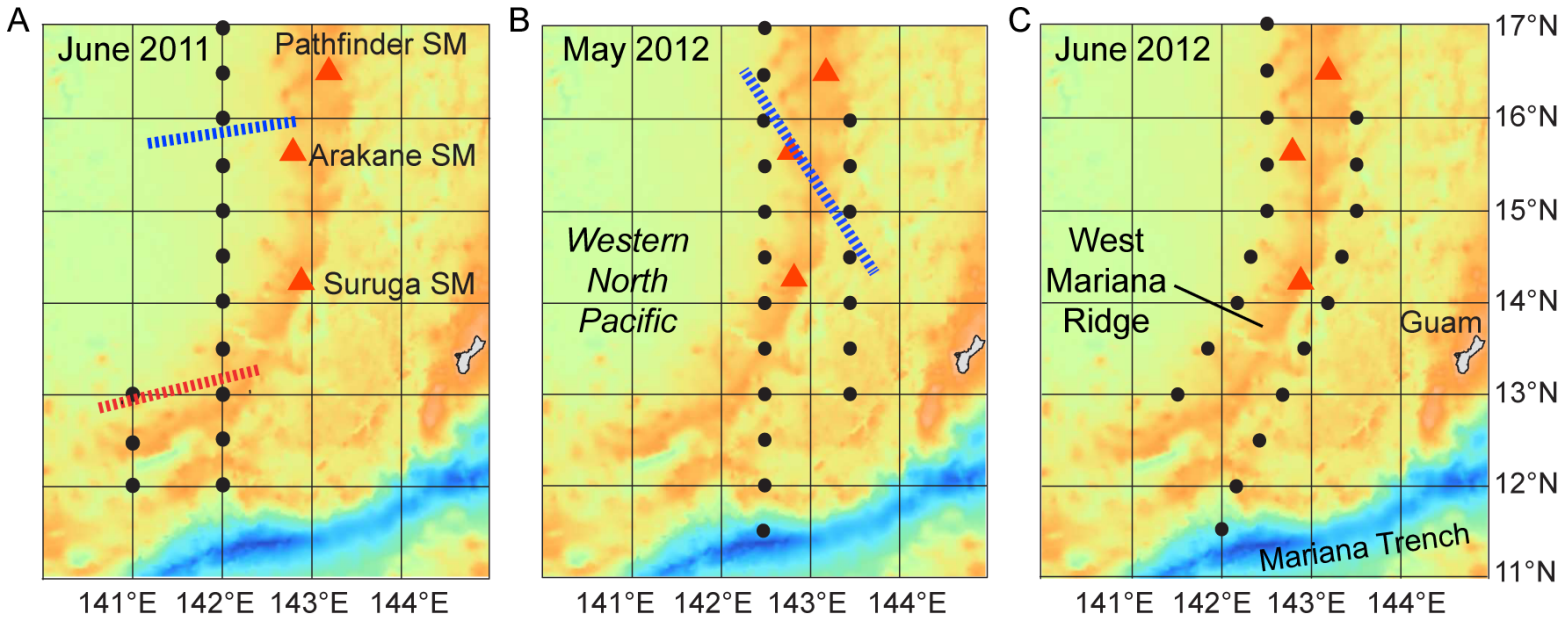
Hydrographic stations along the West Mariana Ridge. The locations of CTD or X-CTD profile stations during the three surveys in 2011 and 2012 are shown with black dots. The estimated location and angle of the salinity front crossing the ridge (red line) and the northern extent of the layer of low salinity water usually associated with the salinity fronts (≤34.5) (blue lines) if present are also shown. Three shallow seamounts (SM) previously investigated are shown (red triangles).

**Figure 3:**
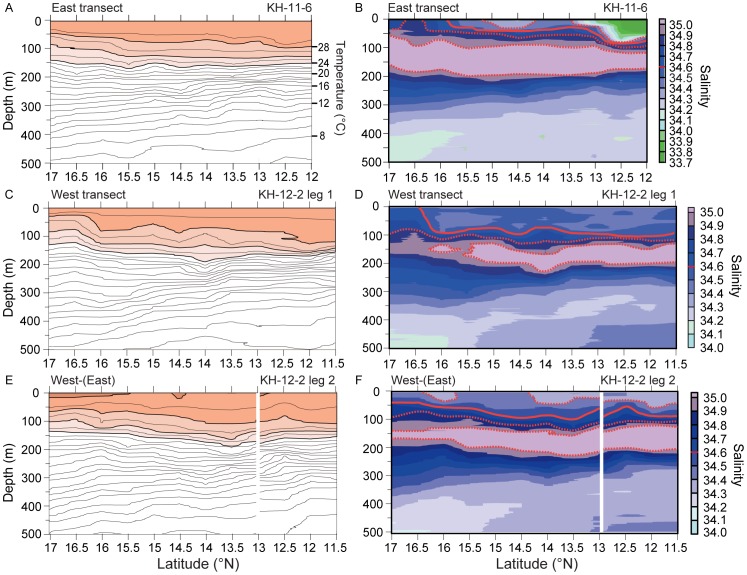
Temperature and salinity structure along the West Mariana Ridge. Hydrographic sections showing the temperature (left panels) and salinity (right panels) structure during the three sampling surveys in 2011 (A, B, June) and 2012 (C, D, May; E, F, June) made using some of the CTD or X-CTD profile stations shown in Figure 2. In the temperature sections, the orange color shows the water in the surface layer <24°C, with contours plotted for every 1°C. In the salinity sections, the solid red lines show the bottom of the 34.5 water, with the 1–2 dotted red lines above or below showing salinity intervals of 0.2 (every second contour), as also shown in Figure 4A,D,G. The southern portion of the eastern transect in E and F was combined with the western transect of that cruise, but the other sections from each cruise are not shown.

Oceanographic observations were made to construct salinity and temperature sections using profiles from either conductivity, temperature, depth (CTD) sensor system (Seabird, USA) deployed from a cable on the side of the ship (to a depth of 500 m depth) or expendable X-CTD probes (Tsurumi Seiki Co. Ltd., Japan) deployed from the back of the slowly moving ship (to a depth of 1000 m depth). Stations were planned east and west of the ridge, but some stations were cancelled on one or the other side of the ridge after the location of the sanity front had been determined (Fig. 2,3).

### Collecting Eggs and Preleptocephali

**Figure 4:**
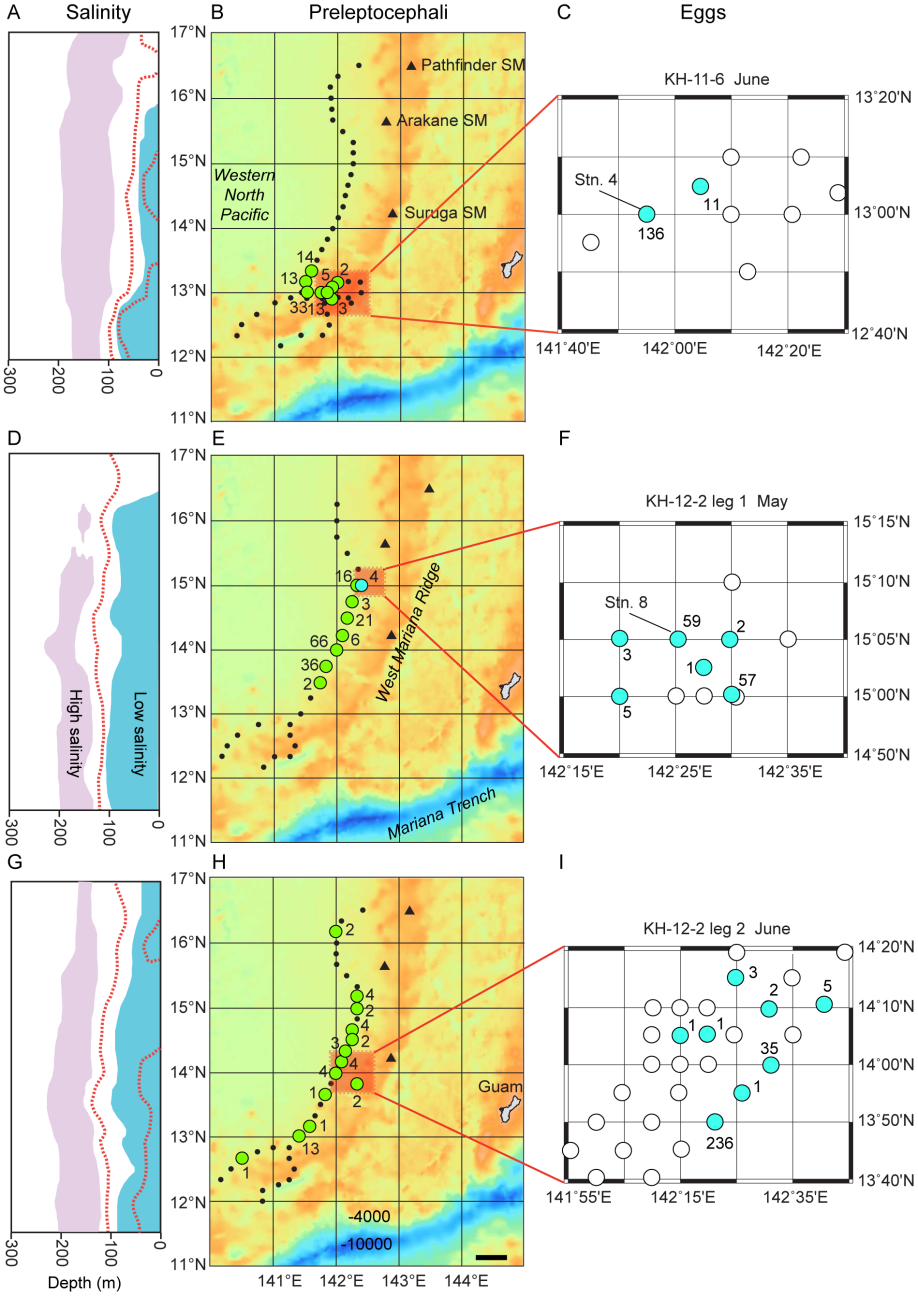
Catches of Japanese eel, *Anguilla japonica*, eggs and preleptocephali in relation to surface salinity structure. Locations where Japanese eel eggs and preleptocephali were collected during R/V *Hakuho Maru* cruises along the West Mariana Ridge in (A–C) June 2011, (D–F) May 2012, and (G–I) June 2012. The latitudes of the salinity structure panels on the left correspond directly to the latitude in the maps in the middle, and the blue-green color in the left panels shows low salinity water ≤34.5, light-purple shows 35.0 salinity, and dotted-red lines show contours spaced at 0.2 salinity intervals above or below the bottom of the ≤34.5 water in the surface layer. In the middle panels, light-green circles show preleptocephalus catches and black dots show no-catch stations, and in the right side insets, light-blue circles show egg catches and white circles show no-catch stations, with numbers of specimens shown with numbers. Four eggs were caught at a station sampled again in the preleptocephali transect (light-blue circle in E). Orange shows shallower depths associated mostly with the West Mariana Ridge and the Mariana Ridge including Guam (middle panels), and blue shows deeper depths down to 10,000 m in the Mariana Trench, which is the deepest place in the world's oceans. Scale bar shows 50 km (H). More than one tow was made at stations 4 and 8 in (C) and (F), respectively.

Sampling to determine the horizontal distributions of fertilized eggs (embryos) and preleptocephali was conducted using standardized oblique tows of a 3-m diameter ORI-BigFish ring net with 0.5 mm mesh that fished mostly in the upper 200 m. Salinity structure was primarily used to decide the starting point of sampling for eggs, and once eggs were found then their distribution was examined in a grid of stations (Fig. 4). After the new moon when the eggs hatched into preleptocephali during all three cruises, transect surveys were carried out over the entire length of the ridge within the spawning area. The stations were at similar distances from the outer ridge, which shifts further to the west and becomes wider with deeper seamounts in the southern region, to determine the possible range of spawning latitudes based on the distribution of preleptocephali (Fig. 4). Most stations had only one ORI-BF tow, but a few had more than one tow. During June 2011, 9 stations were sampled in the egg grid area (including 7 tows in the depth experiment at Stn. 4), followed by 46 stations for preleptocephali after new moon. In May 2012, 8 stations in an overlapping area as the later stations (14.6–16.0°N, not shown) were sampled before new moon prior to sampling in the egg grid, which was followed by 25 stations for preleptocephali. In June 2012, 13 stations (13.5–15.2°N, not shown) were sampled before the egg grid, followed by 37 stations for preleptocephali. For clarity and simplicity, catches of eggs and preleptocephali are presented as number of specimens collected, which is directly understandable, rather than as catch rate values, because their presence or absence is the main factor to consider in this study, not their quantitative relative abundances calculated using the amount of water filtered by the net. The lengths of the preleptocephali (∼4–5 mm) were not evaluated in this study, because these are a short duration pre-feeding stage of larvae, which do not grow until they develop into leptocephali and begin feeding.

For these research surveys, all necessary permits to conduct the biological sampling and hydrographic observations in the Exclusive Economic Zone of the coastal states for both 2011 and 2012 were issued by the United States Department of State, Bureau of Oceans and International Environmental and Scientific Affairs, Department of Foreign Affairs, Federated States of Micronesia and the Ministry of State of the Republic of Palau. No specific animal welfare permit is required in Japan for collecting planktonic organisms.

Eggs (Fig. S1) and preleptocephali (Fig. S2) were sorted out of the plankton samples and morphologically identified before subsamples were genetically confirmed onboard. After being sorted out of the plankton samples, the morphology of the eggs and preleptocephali were examined and photographed using a Nikon SMZ1500 dissecting microscope and a Nikon DMX1200F digital imaging system (Nikon, Tokyo Japan) (Fig. S1, S2). Specimens were then mostly preserved in 99% ethanol to enable later DNA analyses.

### Onboard Genetic Identification

Before deciding to conduct a grid survey around an egg collection location, eggs with the appropriate size of about 1.6 mm and morphology [47] were genetically identified using an onboard real-time polymerase chain reaction (PCR) based ABI PRISM 7300 Sequence Detection System (Applied Biosystems, USA) [48], [49] as described previously [19], [47]. Some preleptocephali were also confirmed onboard to be of the Japanese eel using this system.

### Vertical Distribution of Eggs

**Figure 5:**
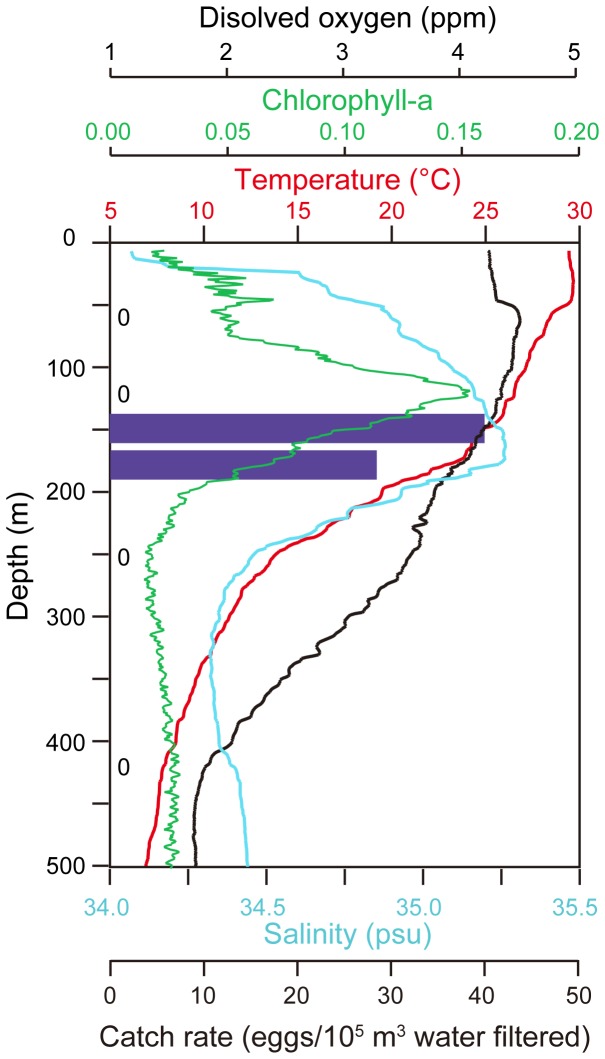
Vertical distribution of Japanese eel, *Anguilla japonica*, eggs. Catch rates of eggs in tows at the 150-axis show the depths of tows that collected no eggs.

During the KH-11-6 survey, the vertical distribution of eggs was studied by making multiple tows at the same station after eggs were collected there. This was conducted on 29 June 2011 at Stn. 4 (13°00N, 141°55′E) (Fig. 4C). The ORI-BF net was deployed in 7 tows that had 20 min of horizontal towing at one of 6 depths (60, 120, 150, 180, 250, 420 m) from 07:38 to 16:20 during the day. The catch rate of eggs in each tow was used as a measure of abundance at each depth layer based on the amount of water filtered by the net calculated from the flow meter revolutions. Two tows were made at 150 m to obtain more eggs for a different study, so the catch rates of the two tows were averaged at that depth in Figure 5. The depth of the net was monitored acoustically with a net depth monitoring system (Scanmar, Norway). The net was open during each tow, but there was no evidence of contamination from other layers, because eggs were only collected at two layers.

## Results

The hydrographic sections of the upper 500 m of the ocean from the three different surveys showed similar general patterns of temperature and salinity structure, except for the patterns of the lower salinity water in the upper 100 m. Water temperatures in the upper 100 m ranged from 26–30°C in the 3 sections, and no temperature fronts were present (Fig. 3A,C,E). The depth of the warmest water in the surface layer became shallower in the north in all three sections, but 30°C water was only detected in June 2012 (Fig. 3E). The patterns of fluctuations of the depth of the isotherms appeared to vary in conjunction with salinity structure in all three pairs of sections in Figure 3. Water with a salinity of 34.5 has been found to be a reliable marker associated with the salinity fronts [16], [39], [40], [46], so this salinity value was used in the present study to show the distribution of salinity levels. Surface water with a salinity of ≤34.5 is shown by red lines in Figure 3B,D,F that correspond to the blue-green shaded water in Figure 4 (left panels). In all three sections the lower salinity water (≤34.5) that is usually associated with the salinity fronts was present in the upper 100 m, but its depth and latitudinal extent varied among surveys (Fig. 3B,D,F). Each hydrographic section showed that the salinity increased with depth until about 75–150 m where there was a layer of high salinity water of various thickness and northerly extension (Fig. 3,4). This subsurface layer of high salinity water is referred to as the North Pacific Tropical Water [40].

In the June 2011 survey there was a shallow layer of lower salinity water ≤34.5 extending almost to 16°N (solid red line in Figure 2B), but there was a distinct salinity front extending deeper between about 12.5 and 13.5°N. Eggs were collected at two stations within the frontal zone (n = 147, 2 days before new moon), and preleptocephali were only collected in the same area (10 stations, n = 83) (Fig. 4A,B,C). No preleptocephali were found either to the north or south of that area after new moon (Fig. 4B).

In leg 1 of the survey in May 2012 a deeper layer of ≤34.5 water extended slighter further to the north compared to in June 2011 and almost reached 16.5°N (Fig. 2D). Eggs were collected in 9 tows at 6 stations near 15°N (n = 128, 0–3 days before new moon, and 4 eggs collected one day after new moon) about 1° south of a possible weak subsurface salinity front associated with the northern limit of the low salinity water (Fig. 4D,E,F). There were 153 preleptocephali collected at 7 stations from 13°30–15°N within the low salinity water after new moon (Fig. 4D,E).

During the next month in the June 2012 leg 2 survey, the layer of lower salinity water ≤34.5 extended all across the study area and to the north of 17°N (Fig. 2F). There was a pool of even lower salinity water ≤34.3 (dotted red line near surface) extending almost to 14.4°N and a separated patch of that water was also detected in the north. Eggs were collected at 8 stations near 14°N at the northern edge of that southern layer of ≤34.3 water (n = 284, 1–3 days before new moon) (Fig. 4G,H,I). Preleptocephali were collected both north and south of the egg collection area, with 2 being caught in the patch of ≤34.3 water in the north, and 1 being caught in the southwest area after new moon (Fig. 4G,H).

The tows made at Stn. 4 to study egg depth distribution in the June 2011 survey collected 21 eggs in two depth layers (150, 180 m), but no eggs were caught in the tows at layers above and below (60, 120, 250, 420 m) as shown in Figure 5. The layer of egg collection was near the top of the thermocline within the center of the high salinity water and just below the chlorophyll maximum, with the most eggs being caught in the 150 m layer.

## Discussion

This study collected both eggs and preleptocephali along the West Mariana Ridge during each of three sampling surveys that were timed in relation to the new moon periods of May or June of 201l and 2012. The eggs were found just before new moon at three different latitudes along the ridge, which corresponded to the locations of the salinity front or being within the latitudinal extent of the low salinity surface water. Egg catches to the south of the strong salinity front in June 2011 were consistent with the hypothesis that the salinity front influences the latitude of spawning [16], [39], [40]. The catch locations of preleptocephali were also limited to the frontal area during that survey. This suggests that spawning eels may have focused on that particular area to form spawning aggregations. The first collections of Japanese eel eggs in May 2009 were also in a similar area just south of a salinity front, with no preleptocephali being collected at stations to the north of the front [19]. Surveys in previous years have found no evidence of spawning on the east side of the ridge [19], which is why there were no stations there during this study.

However, there were no clear salinity fronts in the two 2012 surveys and in some previous studies [17], [41], so the eels must sometimes have to decide where to form spawning aggregations without a distinct salinity front as a landmark. In May and June of 2012 of this study, eggs were collected within the latitudinal range of the low salinity surface water and the catch locations of preleptocephali suggested that spawning had also occurred at a wide range of latitudes. Eddies could redistribute some larvae to slightly different latitudes than those at which they were actually spawned, but in the two 2012 surveys, it is unlikely to account for the broad latitudinal distribution of preleptocephali just a few days after they were spawned. A similar situation was found in 2008 when preleptocephali were collected over almost 2 degrees of latitude within the low salinity water [19], [41]. Although no preleptocephali were collected near the northern edge of the low salinity water in May of 2012, the two surveys in that year suggest that if there is no distinct salinity front, Japanese eels may spawn at multiple latitudes south of the northern limit of the lower salinity surface water. The limited distribution of stations that sampled for preleptocephali was not wide enough in each of the three surveys of this study to determine all places where spawning did not occur, but the collections of preleptocephali were good indications of the general latitudes where spawning likely did occur.

The importance of the salinity front for the Japanese eel was first suggested in 1991 when many leptocephali mostly about 10–20 mm were collected further west in the NEC, just south of a distinct salinity front around 16°N [16]. Similarly, leptocephali were found in waters south of a salinity front in 1994 [50] and in 2002 [39]. The distributions of eggs and preleptocephali revealed in the present study showed that the salinity structure across the spawning area can influence the range of latitudes at which spawning may occur during each new moon period within the spawning season. When a distinct salinity front is present, eels may attempt to aggregate and spawn at or south of the front as occurred in June 2011 and May 2009 [19] even if it is located in the southern part of the NEC (∼12°N). Using a landmark such as a front might facilitate finding mates and increase the successful fertilization of the eggs by having many eels of both sexes involved in the spawning aggregations, rather than just a few as a result of spawning being spread over a wide range of latitudes. Temperature fronts have been hypothesized to have the same function for spawning of Atlantic eels to stop their migration in the Sargasso Sea and look for mates [51], [52].

Although the surveys of this study and previous ones have usually had the problem of not having enough time after new moon to adequately determine the precise spatial extent of spawning by sampling for preleptocephali in multiple gridlines at different longitudes, an interesting pattern has emerged nevertheless. Eggs and preleptocephali have been collected at various latitudes mostly between 12.2–15.3°N on the west side of the West Marina Ridge and not on the east side during multiple years [18], [19], indicating that the seamount chain acts as a landmark of the eastern edge of the spawning area. The location of the salinity front has been linked to the estimated spawning locations in several different years [16], [19], [39] including 2011, but in some years low salinity water extends across the spawning area and no distinct fronts are present [41] as was the case in 2012.

When salinity fronts form within the latitudes of the spawning area, which all types of data suggest is from about 12–16°N [17]–[19], spawning will occur at or south of the front and the eggs will be found along the west side of the seamount ridge. When no front is present within the latitudes of the spawning area, spawning can occur at various latitudes within the lower salinity surface water and eggs may appear in many places along the west side of the ridge. It is unclear if other factors such as differences in current velocities of parts of the NEC may have some influence on the latitude of spawning in the absence of a distinct salinity front, but there has been no clear indication of this occurring.

In the vertical axis of ocean depth, the eggs were only collected at the two layers of 150 and 180 m and not in the 2 shallower and 2 deeper layers. Previous catches of preleptocephali [19], [21] within the same depth layer as the eggs in this study suggest spawning occurs near this depth stratum located at the top of the thermocline. However, since eggs and preleptocephali may be positively buoyant compared to seawater at these approximate temperatures [53], the eggs may rise up until they reach the depth stratum where they seem to accumulate. The development time of Japanese eel eggs at these temperatures are about 1.5–2 days before hatching [54], so they have time to float up to the thermocline. Catches of adult eels in the spawning area in the upper 250 m [19], [21], [22] also suggest that spawning probably occurs at depths of about 160–250 m. Combining all this information, spawning by the Japanese eel seems to be relatively shallow as previously hypothesized [19] and not much deeper as was indirectly suggested by the observation from a submersible of a possible American eel at a depth of about 2000 m on the bottom in the Bahamas far from the American eel spawning area [55]. A possible male Japanese eel was observed 2 days before new moon within the spawning area along the West Mariana Ridge by a underwater camera system “Deep-Tow”(JAMSTEC) at a depth of 179 m in July 2012 [38], but like the possible American eel seen previously, the eel could not be identified for certain.

In the temporal axis, spawning of the Japanese eel appears to be timed to occur in the few days just before new moon. This has been shown by otolith analyses of leptocephali in several different years that showed back-calculated hatching dates were centered only on new moon periods [17], [26]. More direct evidence of new moon spawning has been found by the collections of eggs just before new moon in 2009 [19], and in 2011 and 2012 in this study. Similarly, preleptocephali have only been collected near or just after new moon in 2005 [20], 2007, 2008, 2009 [19] and in this study. Spawning condition adults were also only caught within the spawning area during new moon periods [19], [21], [22].

Therefore, it is hypothesized that Japanese eels spawn just before new moon near or below the top of the thermocline along the seamount ridge, which is determined latitudinally by the northern extent of shallow lower salinity waters. How they determine where these locations are before each new moon period and why these locations would have been established as preferred spawning locations are still unclear though, because little is known about silver eel migrations in the ocean [31]. Pop-up tag studies for the Japanese eel [34] and other anguillid eels [33], [35]–[37] showed that they mostly migrate between depths of about 100–400 m at night (much deeper during the day). However, they can sometimes come into even shallower layers at night [34], [35] at depths close to where they would be able to detect the low salinity surface layers described in this study. There is no evidence that these eels have reached their spawning areas though, so their behaviors might drastically change once they reach the spawning area to include more movements into depths closer to the surface where they could find some aspect of the low salinity water mass, or its absence. Japanese eels and other anguillids are known to have a geomagnetic sense [32], [56], [57] that might be used to help find their spawning areas, but there has also been speculation that they might use various types of odors associated with fronts or different water masses, or pheromones from each other, to locate their spawning sites and to find mates [31], [51], [52]. However, it is essentially still a mystery how the eels reach their spawning areas and find mates for spawning.

Regardless of how the eels accomplish the challenge of reaching their spawning area, for the Japanese eel, the patterns of currents in the western North Pacific have the potential to influence the recruitment success of its larvae. Southward shifts of the spawning locations following El Niño events or shifts of the NEC bifurcation latitude have been hypothesized to increase entrainment of Japanese eel leptocephali into the Mindanao Current and reduce their recruitment to East Asia [39], [40], [43], [45]. Evidence that this can occur was seen when a 42.8 mm *A. japonica* leptocephalus was collected in the Celebes Sea [58] (Fig. 1). The modeling studies showed that a slight difference (1°) in latitudes of spawning can theoretically have a significant effect on the proportions of larvae entering the Kuroshio (chance for successful recruitment) or the southward branch entering the Mindanao Current (recruitment failure) [43], [45]. Other ocean-atmosphere or climatic factors have been also suggested to be related to the recruitment success of the Japanese eel or other species, including productivity changes affecting larval survival [58]–[65]. The detection of unusual temporal patterns of glass eel recruitment has also raised the question about the possibility of there being a shift in the spawning season recently [66]. Which of these factors may be more important in influencing the decline or interannual recruitment fluctuations of the Japanese eel is not known. It is also not known if having a clear landmark to assist Japanese eels to decide where to spawn might improve their spawning and recruitment success, compared to when spawning aggregations are more spread out latitudinally.

Even though some aspects of where and when Japanese eels spawn are now known, many mysteries remain to be determined about how these remarkable fish are able to find their spawning areas and then detect small differences in salinity or other oceanographic characteristics before they form aggregations to spawn. Future efforts to observe spawning aggregations using camera systems [38] may help to understand the reproductive ecology of the Japanese eel, and efforts to learn how their leptocephali are transported westward from different spawning latitudes also needs to be investigated in relation to seasonal patterns of recruitment. These types of information may be important components of the process of finding out how to best manage and conserve this species to help prevent further declines in its population.

## Supporting Information

Figure S1 **Japanese eel,** ***Anguilla japonica*** **, eggs from the spawning area.** Photographs of various stages of freshly caught Japanese eel eggs (embryos) caught along the West Mariana Ridge during the three surveys at the locations shown in Figure 4. Some late-stage embryos hatched out while being observed (A, bottom right; B, right). Many early-stage eggs did not survive the agitation and temperature shock of capture by the net (C, right).
(TIF)

Figure S2 **Japanese eel,** ***Anguilla japonica*** **, preleptocephali from the spawning area.** Photographs of a 5.7 mm early-stage Japanese eel, *Anguilla japonica*, preleptocephalus (pre-feeding stage larva) with a large oil globule and undeveloped head (A), and a 5.0 mm late-stage preleptocephalus with a jaws, teeth, and pigmented eyes, which were both collected at 13°00.1N, 141°24.9E on 21 June 2012 (B). Both larvae were confirmed to be *A. japonica* using onboard Real-Time PCR. Scale bars show 1 mm.
(TIF)
