# Supplementary figures and images for: Spawning Sites of the Japanese Eel in Relation to Oceanographic Structure and the West Mariana Ridge
Source: PLoS One. 2014 Feb 13;9(2):e88759. doi: 10.1371/journal.pone.0088759 (PMC3923831; doi:10.1371/journal.pone.0088759)

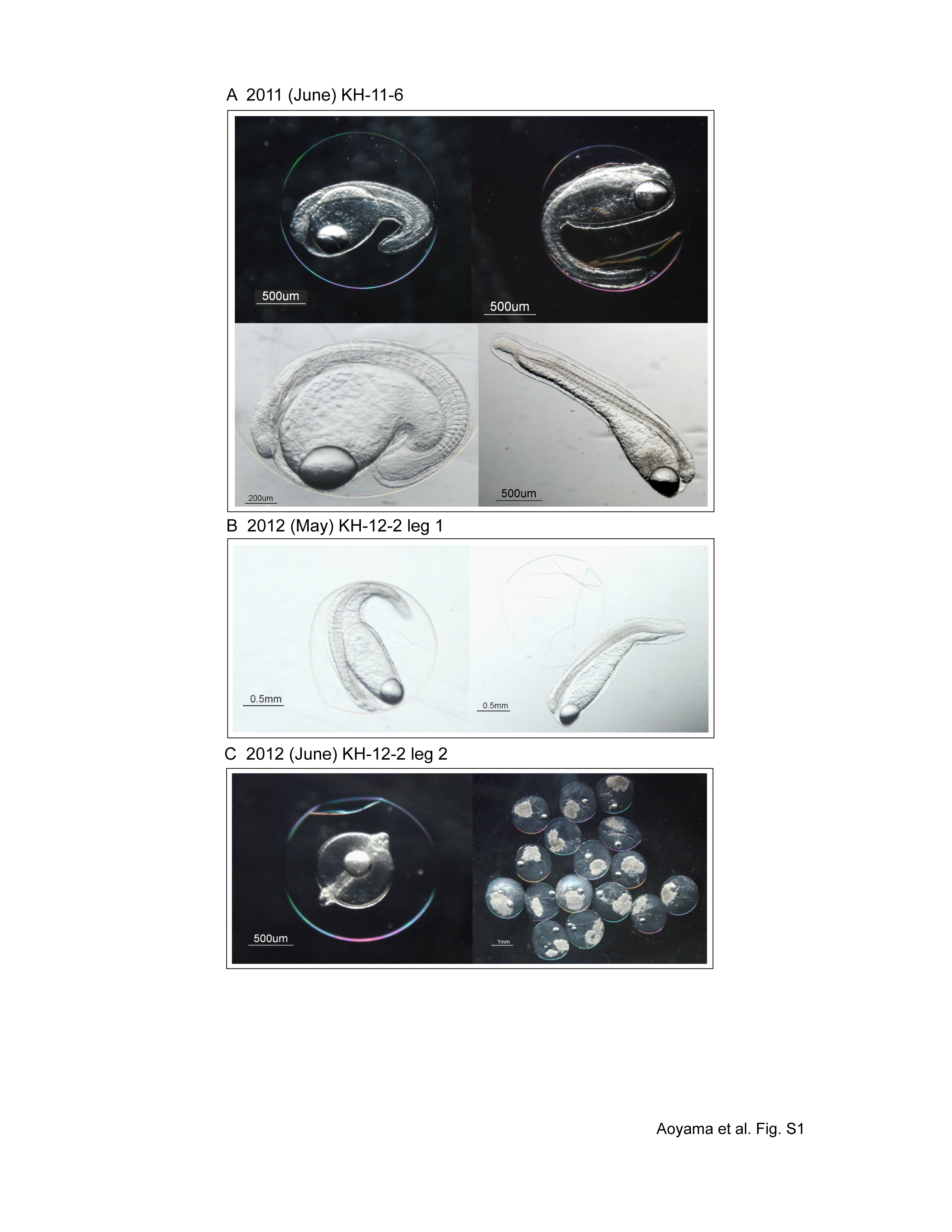

Supplement: Figure S1 — Japanese eel, Anguilla japonica , eggs from the spawning area. Photographs of various stages of freshly caught Japanese eel eggs (embryos) caught along the West Mariana Ridge during the three surveys at the locations shown in Figure 4. Some late-stage embryos hatched out while being observed (A, bottom right; B, right). Many early-stage eggs did not survive the agitation and temperature shock of capture by the net (C, right). (TIF) [file pone.0088759.s001.tif]

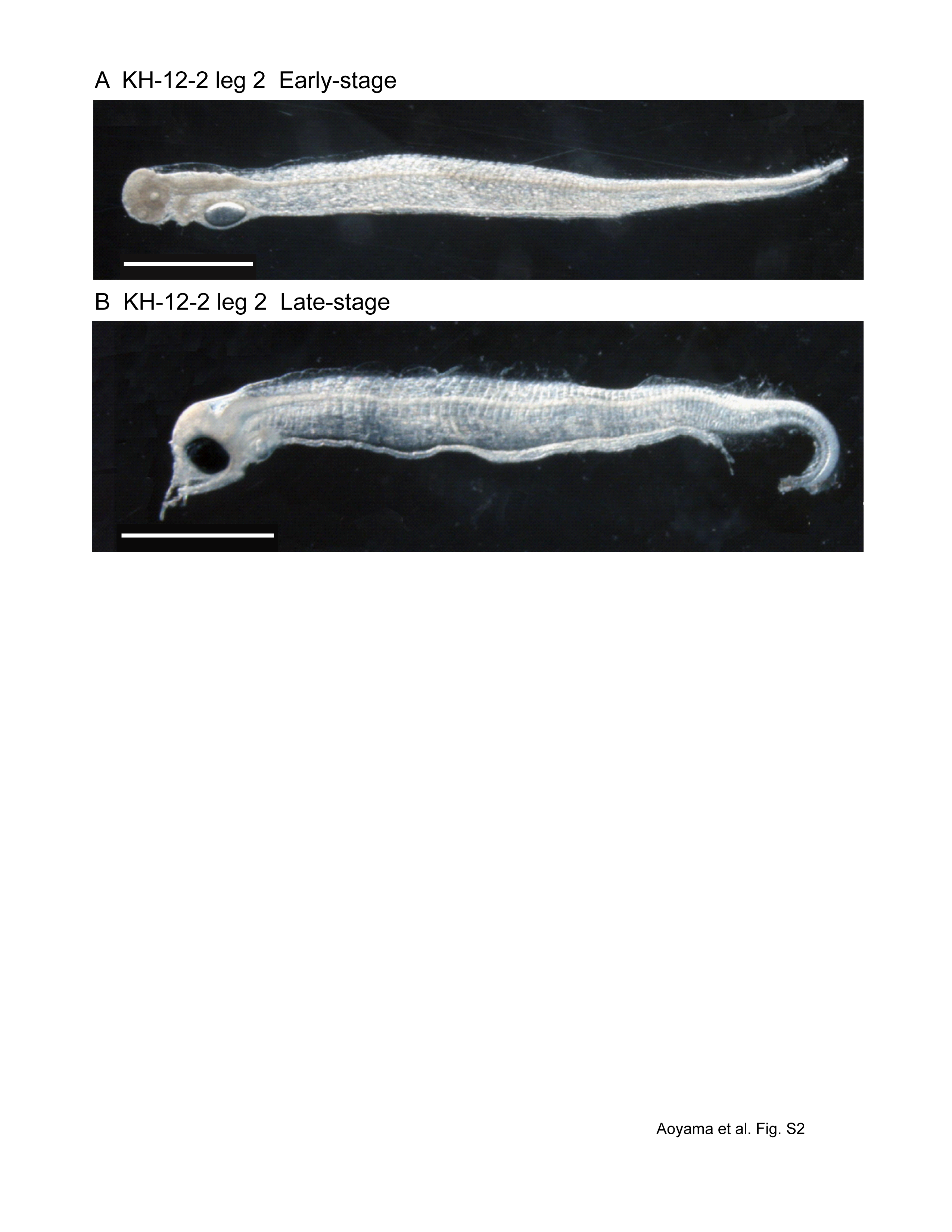

Supplement: Figure S2 — Japanese eel, Anguilla japonica , preleptocephali from the spawning area. Photographs of a 5.7 mm early-stage Japanese eel, Anguilla japonica, preleptocephalus (pre-feeding stage larva) with a large oil globule and undeveloped head (A), and a 5.0 mm late-stage preleptocephalus with a jaws, teeth, and pigmented eyes, which were both collected at 13°00.1N, 141°24.9E on 21 June 2012 (B). Both larvae were confirmed to be A. japonica using onboard Real-Time PCR. Scale bars show 1 mm. (TIF) [file pone.0088759.s002.tif]
